# Metabolic Profiles of Carbohydrates in *Streptococcus thermophilus* During pH-Controlled Batch Fermentation

**DOI:** 10.3389/fmicb.2020.01131

**Published:** 2020-05-29

**Authors:** Gefei Liu, Yali Qiao, Yanjiao Zhang, Cong Leng, Hongyu Chen, Jiahui Sun, Xuejing Fan, Aili Li, Zhen Feng

**Affiliations:** Key Laboratory of Dairy Science, Ministry of Education, College of Food Science, Northeast Agricultural University, Harbin, China

**Keywords:** lactose metabolism, metabolomics, *Streptococcus thermophilus*, transcriptomics, metabolite profiles, gene expression levels profiles

## Abstract

Revealing the metabolic profiles of carbohydrates with their regulatory genes and metabolites is conducive to understanding their mechanism of utilization in *Streptococcus thermophilus* MN-ZLW-002 during pH-controlled batch fermentation. Transcriptomics and metabolomics were used to study carbohydrate metabolism. More than 200 unigenes were involved in carbohydrate transport. Of these unigenes, 55 were involved in the phosphotransferase system (PTS), which had higher expression levels than those involved in ABC protein-dependent systems, permeases, and symporters. The expression levels of the genes involved in the carbohydrate transport systems and phosphate transport system were high at the end-lag and end-exponential growth phases, respectively. In addition, 166 differentially expressed genes (DEGs) associated with carbohydrate metabolism were identified. Most genes had their highest expression levels at the end-lag phase. The *pfk*, *ldh*, *zwf*, and *E3.2.1.21* genes involved in the glycolytic pathway had higher expression levels at the end-exponential growth phase than the mid-exponential growth phase. The results showed high expression levels of *lacZ* and *galKTM genes* and reabsorption of extracellular galactose. *S. thermophilus* MN-ZLW-002 can metabolize and utilize galactose. Overall, this comprehensive network of carbohydrate metabolism is useful for further studies of the control of glycolytic pathway during the high-density culture of *S*. *thermophilus*.

## Introduction

*Streptococcus thermophilus* is widely used as a starter culture in the industrial production of yogurt and cheese (Yong et al., 2011). This bacterium is a fastidious organism that requires carbohydrates, amino acids, vitamins, nucleotides, and minerals for growth in a defined medium. Carbohydrates are the main source of energy for bacterial growth and can be metabolized by lactic acid bacteria (LAB) via either homofermentative or heterofermentative fermentation. *S. thermophilus* ferment sugars by the Embden-Meyerhoff-Parnas (EMP) pathway to pyruvate, which is converted into lactic acid by lactate dehydrogenase (LDH). Mixed-acid metabolism, a type of homofermentation, is characterized by the production of formate, acetate, ethanol, and/or CO_2_ in addition to lactate. And the homolactic metabolism can be shifted to a mixed-acid metabolism under certain conditions (carbon limitation, carbon excess of slowly metabolized sugars) (Mayo et al., 2015). However, the mechanisms underlying the shift from homolactic fermentation to mixed-acid fermentation have been the subject of considerable controversy (Papagianni et al., 2007). The primary metabolites and genes of carbohydrate metabolism in LAB have been studied. However, the specific details of the accumulation, reabsorption, and reuse of the intermediate metabolites of carbohydrate metabolism during the culture of the LAB have not been thoroughly elucidated to date. In addition, specific changes in the expression levels of the genes involved in carbohydrate metabolism remain unclear.

*Streptococcus thermophilus* can metabolize a variety of carbohydrates but prefers lactose. *S. thermophilus* can rapidly convert lactose into lactic acid through the glycolytic pathway and can also produce chemicals that affect the final texture and flavor of the product, such as acetic acid, acetaldehyde, formic acid, and diacetyl (Rasmussen et al., 2008). Starter lactococcal strains transport lactose into the cell using the LacS permease and the phosphoenolpyruvate-dependent phosphotransferase system (PEP-PTS) (Barrangou et al., 2006; Jojima et al., 2010; Aleksandrzak-Piekarczyk, 2013). After translocation via lac-phosphotransferase system (PTS), lactose is hydrolyzed by 6-P-β-galactosidase to glucose (metabolized via the glycolytic pathway) and galactose-6-P (metabolized via the D-tagatose-6-P pathway) or hydrolyzed to glucose and galactose (metabolized via the Leloir pathway) (Vaillancourt et al., 2008; Aleksandrzak-Piekarczyk, 2013).

The co-utilization of carbohydrates in bacteria is a substantial research topic of metabolic engineering, which is a way to decrease the formation of byproducts, improve target product yields and reduce microbial production costs (Wu et al., 2016). Understanding the intracellular metabolic mechanism of carbohydrates in *S. thermophilus* can be helpful for optimizing the parameters of the fermentation process to obtain a high-quality starter. In addition, the carbohydrate transport system and glycolytic key enzymes could regulate glycolysis to a certain degree. Thus, understanding the information of the specific changes in the expression levels of the genes involved in carbohydrate metabolism can provide the basic data for these researches.

In this study, changes in the gene transcription levels and the profiles of intracellular and extracellular carbohydrate metabolites were revealed by transcriptomics and metabolomics in *S. thermophilus* MN-ZLW-002 (ST-MZ-2) during pH-controlled batch fermentation, which has been used as a commercial strain for starter production. The overall aim is to provide basic data for the accumulation, reabsorption, and reuse of the intermediate metabolites of carbohydrate metabolism in *S. thermophiles* and theoretical support for the conversion from homofermentation to mixed-acid fermentation by metabolic regulation during the high cell-density culture of *S. thermophilus*.

## Materials and Methods

### Strains, Culture Conditions, and Fermentation Experiments

**FIGURE 1:**
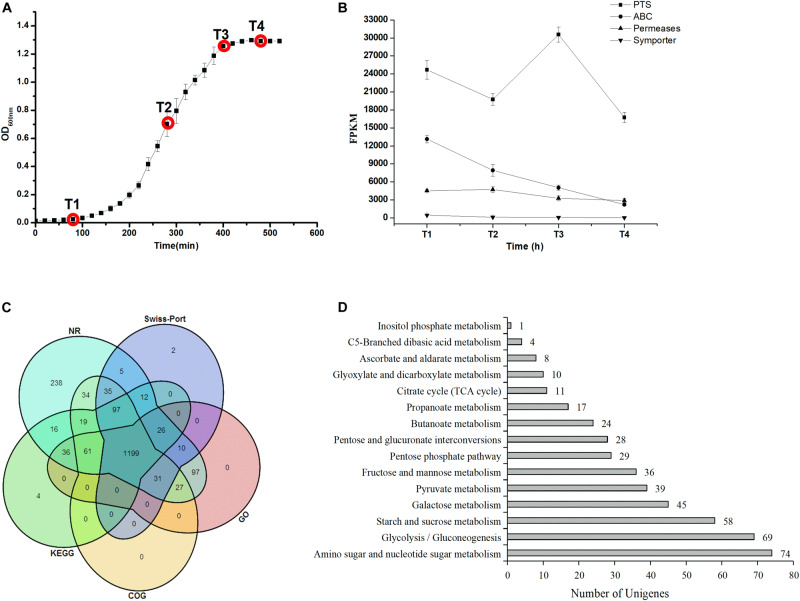
**(A)** Growth curve and sampling points during the ST-MZ-2 culture. **(B)** Changes in the FPKM of four carbohydrate transport systems during ST-MZ-2 culture. **(C)** Venn diagram of the unigenes annotated to the NR, Swiss-Prot, KEGG, COG, and GO. **(D)** The numbers of unigenes annotated to carbohydrate metabolic pathways.

*Streptococcus thermophilus* MN-ZLW-002 was obtained as previously described (Xiaohong et al., 2012). Culture stocks were prepared in 10% (w/v) sterile reconstituted skim milk containing 10% glycerol and stored at −80°C. Before use, three subcultivation steps were performed in a chemically defined medium (CDM) to obtain a stable growth response for 12 h at 42.5°C. The CDM was adapted from that described by Letort and Juillard (2001). Lactose was the carbon source, and the concentration was 10 g/L. Composition of the CDM was listed in Supplementary Table S2. Batch fermentations were performed in a 10-L Biotech-7000 bioreactor (Shanghai Baoxing, Shanghai, China) containing 7 L of CDM. The culture was centrifuged (10,000 × *g*, 10 min, 4°C), and the cells were washed twice with phosphate-buffered saline (PBS) (50 mM, pH 6.5) and inoculated into the bioreactor. The temperature and rotation speed were fixed to 42.5°C and 200 rev/min, respectively. The pH was maintained at 6.25 by the automatic addition of 1 M NaOH. Microaerophilic conditions (no aeration) were applied in this study. The sampling points are shown in Figure 1A. The cultures were centrifuged (12,000 × *g*, 4°C, 15 min) at the time points indicated, and the supernatant and pellet were snap-frozen in liquid nitrogen and stored at −80°C until further analysis, respectively. Each culture condition was repeated three times.

### Transcriptomic Analysis

The RNA isolation and library construction for transcriptome analysis were performed as described by Zeng et al. (2015) with some modifications. The total RNA quantity and purity were assessed using a Bioanalyzer 2100 and RNA 6000 Nano LabChip Kit (Agilent, Santa Clara, CA, United States) with an RNA integrity number of 7.0. Approximately 5 μg of total RNA was used to deplete the ribosomal RNA using the Ribo-Zero Gold rRNA Removal Kit (Illumina, San Diego, CA, United States) according to the manufacturer’s instructions. After removing rRNA, the remaining RNA was reverse-transcribed to generate cDNA, which was then employed to synthesize U-labeled second-stranded DNA using DNA polymerase I, RNase H, and dUTP. An A-base was added to the blunt ends of each strand to facilitate their ligation to the indexed adapters, which had T-base overhangs. Single- or dual-index adapters were ligated to the DNA molecules. After heat-labile UDG enzyme treatment of the U-labeled second-stranded DNA, the ligated products were PCR amplified under the following conditions: initial denaturation at 95°C for 3 min, followed by eight cycles of denaturation at 98°C for 15 s, annealing at 60°C for 15 s, and extension at 72°C for 30 s, with a final extension at 72°C for 5 min. The cDNA was gel purified and ethanol precipitated to generate a more concentrated cDNA library. The average insert size for the final single-end cDNA libraries was 250 bp (±50 bp). Single-read sequencing (50 bp) was performed using an Illumina HiSeq 2500 following the manufacturer’s instructions. The transcriptome data were analyzed as described by Shen et al. (2015) with the exception that clean reads with a length of 36 nt were obtained.

### Metabolomic Analysis

The supernatant and the cell pellets (described in section “Strains, Culture Conditions, and Fermentation Experiments”) were used for the analysis of extracellular metabolites and intracellular metabolites. The supernatant and the cell pellets were frozen for 0.5 h at −20°C, and then allowed to thaw at 4°C. The supernatant was used for the analysis of extracellular metabolites. To extract the intracellular metabolites, the pellet (25 mg) was resuspended in 800 μL of methanol and water (1:1, v/v), and the metabolites were extracted using a TissueLyser (Shanghai Jingxin, Shanghai, China) (60 Hz) for 5 min at room temperature. The cell debris was removed by centrifugation (25,000 × *g*, 4°C, 20 min), and the samples (200 μL) were dried in a vacuum concentrator until the volume was less than 10 μL and resuspended in ultrapure H_2_O to a final volume of 50 μL. One microliter of 0.5 mM AZT was added as an internal standard. Liquid chromatography tandem mass spectrometry data were acquired using a 2777C UPLC system (Waters, United States) coupled to a Xevo G2-XS Q-TOF mass spectrometer (Waters, United States). First, all of the chromatographic separations were performed using an ultra-performance liquid chromatography (UPLC) system (Waters, United States), and an ACQUITY UPLC BEH C18 column (100 mm^∗^2.1 mm, 1.7 μm, Waters, United States) was used for the reversed-phase separation. The column oven was maintained at 50°C, and the flow rate was 0.4 mL/min. The mobile phases consisted of water (A) and acetonitrile (B) that both contained 0.1% formic acid. The elution program was as follows: 0–2 min, 100% phase A; 2–11 min, 0–100% B; 11–13 min, 100% B, and 13–15 min, 100% A. The injection volume was 10 μL.

A high-resolution tandem mass spectrometer (SYNAPT G2 XS Q-TOF, Waters, United States) was used to detect the metabolites that eluted from the column. The Q-TOF was operated in both the positive and negative ion modes. For the positive ion mode, the capillary and sampling cone voltages were set to 0.25 kV and 40 V, respectively. For the negative ion mode, the capillary and sampling cone voltages were set to 2 kV and 40 V, respectively. The mass spectrometry data were acquired in the centroid MSE mode. The TOF mass ranged from 50 to 1200 Da, and the scan time was 0.2 s. For the MS/MS detection, all of the precursors were fragmented with 20–40 eV, and the scan time was 0.2 s. During the acquisition, the LE signal was acquired every 3 s to calibrate the mass accuracy. In addition, to evaluate the stability of the LC-MS instrument during the acquisition, a quality control sample was acquired every 10 samples. The data were processed using Progenesis QI (version 2.2). These analyses were performed in triplicate.

### Function Elucidation and Metabolic Network Construction

The KEGG^1^ and COG databases^2^ were used to classify and group the genes and metabolites identified.

### Transcriptional Analysis by Quantitative Reverse Transcription PCR (qRT-PCR)

Quantitative reverse transcription PCR (qRT-PCR) was performed to determine the expression level of gene associated with carbohydrate metabolism. Total RNA was isolated via the hot phenol method using the TRK-1002 Kit (LC Bio, China). The resulting cDNA was stored at −20°C until qRT-PCR was performed. qRT-PCR was performed with RNA isolated from *S. thermophilus* MN-ZLW-002 at four different growth phases. Primers used for qRT-PCR were listed in Supplementary Table S1. qRT-PCR was carried out in 96-well plates using the ABI StepOnePlus PCR system (American). The 16S rRNA gene was used as an internal control to normalize cycle threshold (CT) values. The 2^–(ΔΔCT)^ method was used to assess the differences in the expression levels of sRNA genes. Three technical replicates were performed for each sample.

## Results

### Analysis of Carbohydrate Metabolism

The genes were identified at four sampling points *de novo* transcriptome during the growth of ST-MZ-2 (Figure 1A). Approximately 308 genes were annotated to 15 carbohydrate metabolic pathways (Figures 1C,D). The genes associated with amino sugar and nucleotide sugar metabolism (ko00520), glycolysis/gluconeogenesis (ko00010), galactose metabolism (ko00052), and pyruvate metabolism (ko00620) were more abundant than those involved in other types of carbohydrate metabolism (Figure 1D). We performed transcriptome profiling at four different growth phases to identify the differentially expressed genes (DEGs) during the growth of ST-MZ-2. In addition, the false discovery rate (FDR) (≦0.001) and absolute value of the log_2_ratio (≧1) were used as analytical conditions to identify DEGs. DGEs of different comparisons for four different growth phases were identified. In addition, 166 DGEs associated with carbohydrate metabolism were identified during the growth of ST-MZ-2.

### Changes in the Expression Levels of the Genes Involved in Carbohydrate Transport Systems

The changes in the sum of the fragments per kilobase of transcript per million fragments mapped (FPKM) involved in carbohydrate transport systems are shown in Figure 1B. More than 200 unigenes were involved in carbohydrates transport during the growth of ST-MZ-2, and 55 of these unigenes were involved in the PTS. The expression levels of the genes associated with the transport systems decreased with the culture time with the exception of PTS. The expression levels of the genes associated with PTS were high at T1 and T3. The expression ratio values of the genes between different times are shown in Figure 3. The results showed that almost half of the genes were downregulated. The expression levels of *crr* encoding PTS-Glc-EIIA associated with glucose transport were high at T1. *LacS* encoding the lactose transporter was downregulated 1.81-fold at T2 compared with T1. *ManX*, *ulaC*, and *fruA* involved in the transport of mannose, ascorbate, and fructose were upregulated from T1 to T3. *FruB* and *gatC* associated with fructose and galactitol transport were upregulated. The majority of the genes associated with PTS were significantly downregulated at T4 with the exception of *gatC* and *fruB*.

### Changes in the Expression Levels of the Genes in the Galactose Metabolic Pathway

**FIGURE 2:**
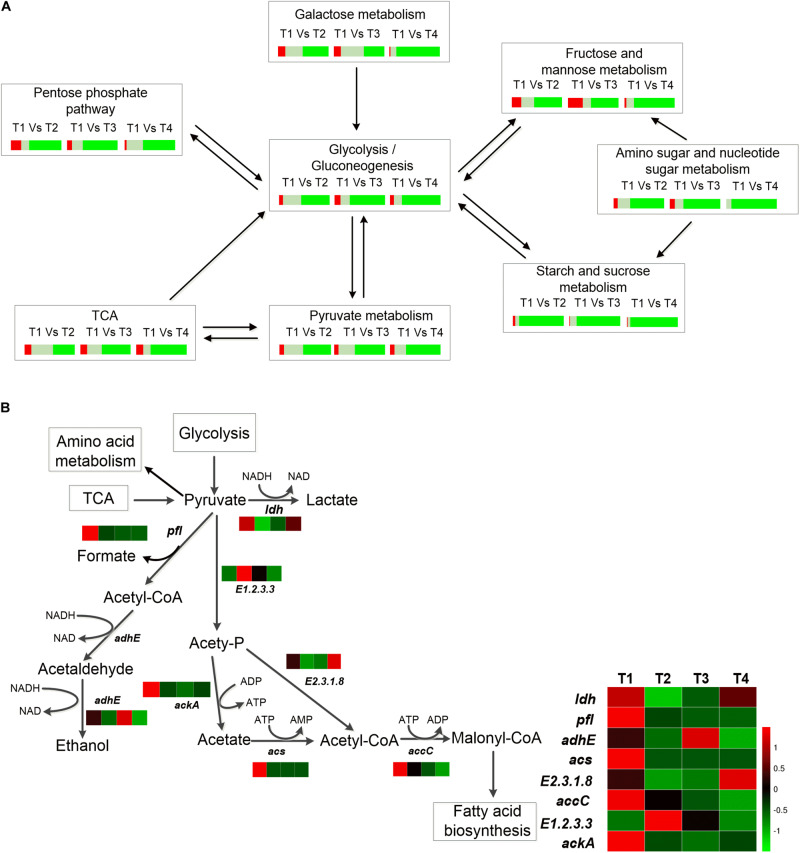
**(A)** Percentage of DEGs of different carbohydrate metabolic pathways. Red represents the percentage of significant upregulated genes (log_2_ratio≧1); gray represents the percentage of insignificant expression genes (-1<log_2_ratio<1), and green represents percentage of significant downregulated genes (log_2_ratio≦−1). **(B)** Heat map of the DEGs of pyruvate metabolism of ST-MZ-2 during culture.

The genes and metabolites associated with carbohydrate metabolism were identified. Simultaneously, the comparison of the gene expression levels between different times was conducted. In general, the number of genes downregulated gradually increased with culture time, except for the genes associated with the metabolism of amino sugar and nucleotide sugar (Figure 2A).

**FIGURE 3:**
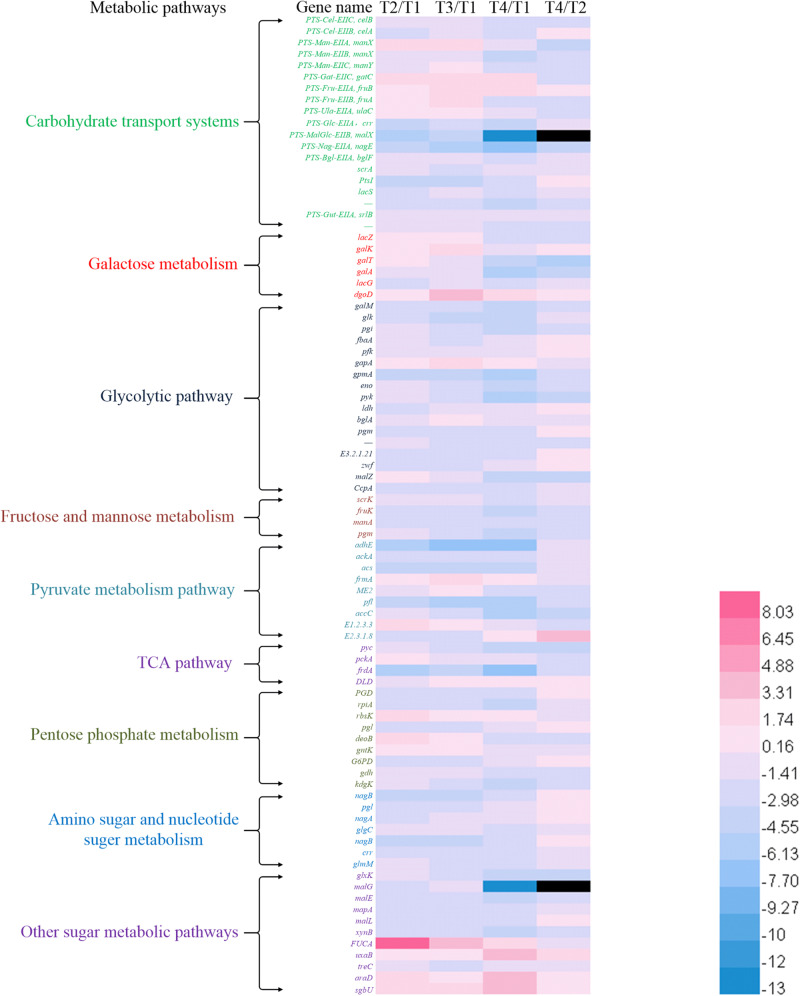
Heat map of expression ratio values of the genes involved in metabolic pathways between different culture times.

The total number of the genes upregulated involved in galactose metabolism did not differ significantly at T2 and T3 compared with T1. In addition, more than 80% of the genes associated with galactose metabolism were downregulated at T4 (Figure 2A). The expression levels of *lacZ* (encoding β-galactosidase) and *galA* (encoding α-galactosidase) were downregulated 1.43-fold and 4.84-fold at T4 compared with T1, respectively (Figure 3). The expression level of *lacS* was also downregulated 2.58-fold at T4 compared with T1 (Figure 3). The expression levels of the genes encoding lactose phosphotransferase, lactose transferase, and *lacG* were high at T1 and T3 (Figures 1B, 3). In the Leloir pathway, *galK* (encoding galactokinase) and *galT* (encoding UDP glucose-hexose-1-phosphate-uridylyltransferase) were key genes. The expression level of *galK* was upregulated from T1 to T3 (Figure 3). The expression level of *galT* was the highest at T2 (Figure 3).

### Changes in the Expression Levels of the Genes in the Glycolytic Pathway

**FIGURE 4:**
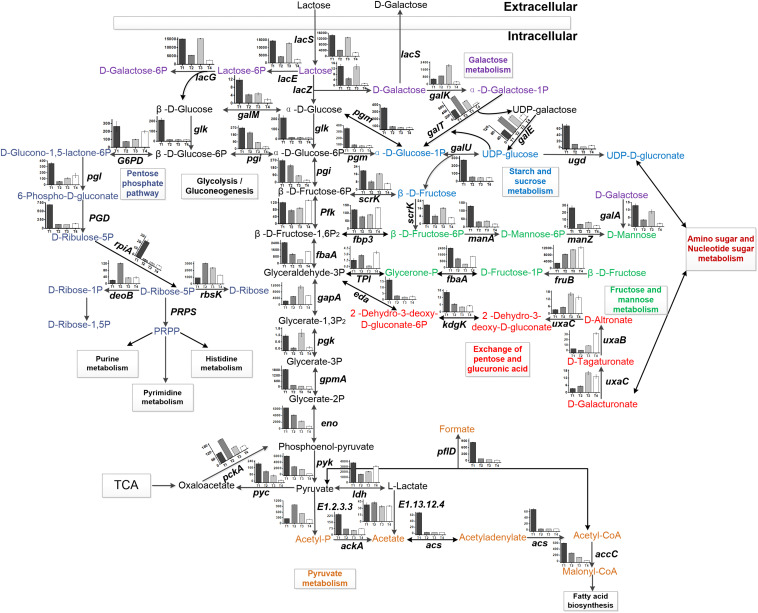
Central carbon metabolism of ST-MZ-2. The histogram shows the FPKM of the genes involved in carbohydrate metabolism at four sampling points during the growth of ST-MZ-2.

The expression levels of the genes associated with the glycolytic pathway are shown in Figure 3. The expression levels of most of the genes decreased gradually with culture time. The expression level of *gapA* encoding glyceraldehyde-3-phosphate dehydrogenase (GADPH) was upregulated from T1 to T3. The total number of the genes downregulated increased gradually from T2 to T4. The expression level of *pfk* (encoding phosphofructokinase) was the lowest at T2. The expression level of *ldh* was the highest at T1 and the lowest at T2. The expression level of *fbaA* encoding fructose-bisphosphate aldolase was downregulated 2.38-fold at T3 compared with T1. *Glk* (encoding glucokinase) and *pyk* [encoding pyruvate kinase (PYK)] were the key genes in the EMP pathway. The expression levels of *glk* and *pyk* were the highest at T1. In addition, glucose-6-phosphate isomerase (encoded by *pgi*) was the key enzyme between the glycolytic and pentose phosphate pathways (Figure 4). Phosphoglucomutase (encoded by *pgm*) was the key enzyme that connected the amino sugar metabolism and the glycolytic pathway (Figure 4). The expression levels of *pgi* and *pgm* were downregulated from T2 to T4 compared with T1. More than 10% of the genes were regulated by carbon catabolite repression (CCR) system. Catabolite control protein A (CcpA), the key protein in the CCR system, was involved in the regulation of the glycolytic pathway. The expression level of *CcpA* was the highest at T1 (Figure 3).

### Changes in the Expression Levels of the Genes in Pyruvate Metabolism and the TCA Pathway

*Streptococcus thermophilus* is a typical homofermentative organism but also produces other metabolites, such as acetic acid, formic acid, acetaldehyde, and ethanol. The expression levels of *pfl* (encoding pyruvate-formate lyase), *adhE* (encoding acetaldehyde dehydrogenase/ethanol dehydrogenase), *ackA* (encoding acetate kinase), *acs* (encoding acetyl-CoA synthase), *accC* (encoding acetyl-CoA carboxylase), and *E2.3.1.8* (encoding phosphate acetyltransferase) were the highest at T1 (Figures 2B, 3). Their expression levels were significantly downregulated at T2 and maintained the low expression levels at T3 and T4 with the exception of *E2.3.1.8*. In contrast, *E2.3.1.8* was upregulated 3.5-fold at T4 compared with T2. The expression level of *E1.2.3.3* (encoding pyruvate oxidase) was significantly upregulated 1.96-fold at T2 compared with T1.

In the TCA pathway, *pyc*, *pckA*, *frdA*, and *DLD* were DEGs. Pyruvate carboxylase (encoded by *pyc*) and phosphoenolpyruvate (PEP) carboxykinase (encoded by *pckA*) were the key enzymes that formed a metabolic cycle between pyruvate metabolism and the TCA pathway (Figure 4). The expression level of *pyc* decreased gradually with culture time and was downregulated more than fourfold at T4 compared with T1 (Figure 3). However, the expression level of *pckA* was the highest at T2. The expression level of *frdA* (encoding fumarate reductase flavoprotein subunit) was downregulated 4.61-fold, 4.48-fold, and 6.38-fold at T2, T3, and T4 compared with T1, respectively. The expression level of *DLD* (encoding dihydrolipoamide dehydrogenase) was the lowest at T2.

### Changes in the Concentrations of Carbohydrate and Its Metabolites

The changes in the concentrations of carbohydrate and its metabolites are shown in Table 1. For extracellular carbohydrate and its metabolites, lactose concentration decreased significantly (*P* < 0.05) with the increase in the concentrations of lactic acid, ethanol, and trehalose. The concentration of galactose was the highest and the lowest at T2 and T3, respectively. In addition, the concentration of galactose had no obvious difference at T2 and T4. The trends of the changes in the concentrations of the other metabolites were similar. These metabolites reached maximal concentrations at T2 and decreased significantly from T3 to T4. The concentration of ethanol was the lowest, and lactic acid was the highest among these metabolites.

**TABLE 1:** Changes in the concentrations of carbohydrates and their metabolites during ST-MZ-2 culture.

**Carbohydrates and their metabolites**	**Abundance of carbohydrates and metabolites (peak area/1000)**			
	
	**FL_T1**	**FL_T2**	**FL_T3**	**FL_T4**
**Extracellular compounds**				
Lactose	555.80 ± 1.42^d^	305.34 ± 9.14^c^	42.46 ± 1.63^b^	1.0 ± 0.63^a^
Galactose	37.39 ± 7.73^a^	75.78 ± 2.05^b^	34.71 ± 6.56^a^	70.94 ± 8.83^b^
Acetic acid	85.05 ± 4.1^a^	165.90 ± 10.68^b^	189.02 ± 3.95^c^	212.52 ± 5.36^d^
Acetaldehyde	13.93 ± 2.72^a^	21.04 ± 5.11^b^	18.15 ± 1.28^ab^	12.71 ± 1.43^a^
Ethanol	0.59 ± 0.07^a^	1.34 ± 0.02^b^	3.15 ± 0.372^c^	6.3 ± 0.531^d^
Lactic acid	6153.76 ± 80.86^c^	9521.95 ± 19.17^d^	5423.20 ± 176.29^b^	3743.36 ± 66.14^a^
D-glucose	27.39 ± 1.16^b^	55.78 ± 2.05^c^	25.37 ± 1.19^b^	2.61 ± 0.36^a^
Trehalose	3.33 ± 0.25^a^	174.23 ± 6.35^b^	207.92 ± 7.18^c^	263.03 ± 15.51^d^
*N*-acetyl-D-galactosamine	50.11 ± 3.3^b^	85.68 ± 2.32^d^	65.17 ± 1.59^c^	13.48 ± 1.26^a^
**Intracellular compounds**				
Lactose	9.11 ± 1.05^a^	41.06 ± 0.83^c^	44.51 ± 1.46^d^	22.20 ± 0.29^b^
Galactose	43.05 ± 1.38^a^	49.49 ± 4.11^ab^	58.18 ± 3.23^b^	79.29 ± 7.88^c^
Acetic acid	24.0 ± 1.94^a^	76.03 ± 4.72^c^	45.09 ± 1.67^b^	24.50 ± 2.9^a^
Acetaldehyde	21.37 ± 3.82^a^	26.35 ± 3.26^ab^	40.37 ± 3.92^c^	31.98 ± 3.73^b^
Ethanol	6.65 ± 2.14^ab^	6.86 ± 3.61^ab^	4.09 ± 3.04^a^	13.25 ± 5.83^b^
Lactic acid	4915.26 ± 71.49^c^	6198.78 ± 88.4^d^	3481.39 ± 121.2^a^	4112.40 ± 63.8^b^
α-D-glucose	72.67 ± 3.31^c^	112.43 ± 3.11^d^	54.89 ± 10.31^b^	38.8 ± 4.7^a^
β-D-fructose	60.2 ± 5.92^a^	103.78 ± 12.28^b^	57.92 ± 8.51^a^	44.02 ± 8.55^a^
Fructose-1-phosphate	17.32 ± 5.26^a^	17.08 ± 0.95^a^	22.93 ± 2.59^ab^	23.86 ± 2.39^b^
5-Phosphoribosyl-1-pyrophosphate	37.57 ± 2.51^a^	47.11 ± 6.3^a^	46.90 ± 5.06^a^	42.97 ± 5.52^a^
2-Deoxy-D-ribose-5-phosphate	29.42 ± 2.60^a^	32.87 ± 4.69^ab^	36.58 ± 0.89^b^	49.85 ± 2.85^c^
2-Deoxy-D-ribose-1-phosphate				
Ribitol	3734.74 ± 7.63^c^	4070.17 ± 73.75^d^	2027.92 ± 71.14^b^	159.23 ± 26.88^a^
Sorbose Mannose	44.31 ± 2.39^a^	50.53 ± 3.77^ab^	59.68 ± 2.55^b^	81.31 ± 8.99^c^
Sucrose Trehalose	11.91 ± 1.75^a^	697.21 ± 27.95^d^	388.14 ± 17.48^c^	53.60 ± 1.23^b^
*N*-acetyl-D-galactosamine	50.77 ± 4.19^a^	64.35 ± 11.2^ab^	66.92 ± 5.47^b^	76.23 ± 6.24^b^
D-Tagatose	44.31 ± 2.39^a^	50.53 ± 3.77^b^	59.68 ± 2.55^c^	81.31 ± 8.99^d^
Riboflavin-5-phosphate	7.52 ± 0.44^a^	84.92 ± 3.48^b^	115.82 ± 9.6^c^	12.10 ± 0.97^a^
Maltose	111.87 ± 4.16^a^	1075.08 ± 70.6^b^	9640.23 ± 102^d^	7190.13 ± 206^c^
Citric acid	2690.24 ± 126.9^d^	204.22 ± 4.65^a^	806.50 ± 12.16^c^	375.94 ± 4.32^b^
Succinate semialdehyde-thiamin diphosphate	47.32 ± 4.11^a^	90.81 ± 8.54^b^	143.03 ± 11.18^c^	153.36 ± 5.83^c^

*Different letters represent significant differences (*P* < 0.05), and the same letters represent that the difference is not significant (*P* > 0.05).*

For intracellular carbohydrate and its metabolites, the concentrations of lactose, acetaldehyde, riboflavin-5-phosphate, and maltose increased significantly from T1 to T3 and decreased at T4. The concentrations of galactose, 2-deoxy-D-ribose-5-phosphate, 2-deoxy-D-ribose-1-phosphate, sorbose, mannose, *N*-acetyl-D-galactosamine, D-tagatose, and succinate semialdehyde-thiamin diphosphate increased gradually with culture time. Acetic acid, α-D-glucose, β-D-fructose, 5-phosphoribosyl-1-pyrophosphate, ribitol, and trehalose reached maximal concentrations at T2 and decreased significantly from T3 to T4. The concentrations of ethanol and lactic acid increased from T1 to T2, decreased from T2 to T3, and increased at T4. The concentration of ethanol was the lowest among these metabolites. However, the concentrations of lactic acid, ribitol, maltose, and citric acid were much higher than those of the other metabolites.

### Validation of Gene Expression Pattern by Using Real-Time Quantitative Reverse Transcription PCR (qRT-PCR)

Validation of gene expression pattern by using real-time qRT-PCR. We chose 12 targeted genes (*pgm*, *pyk*, *ldh*, *galTK*, *pgi*, *lacZS*, *pfk*, *glk*, *pyc*, *CcpA*) that involved in carbohydrate metabolism for qRT-PCR measurements, and the changes of expression were all confirmed. As shown in Supplementary Figure S1, the fold changes for gene regulation predicated from *de novo* transcriptome and qRT-PCR showed a similar or nearly synchronized trend.

## Discussion

The uptake of lactose is regulated by several transport systems, including PTS, ABC protein-dependent systems, permeases, symporters, and glycoside-pentoside-hexuronide transporters (Aleksandrzak-Piekarczyk, 2013; Kowalczyk et al., 2015). The genes involved in carbohydrate transport had high expression levels at the lag phase showing that that the translocation of carbohydrates into cell occurred primarily at the lag phase. Changes in concentrations of intracellular lactose, galactose, and glucose also fully proved this. The results showed that the genes involved in PTS outnumbered the others during the culture of ST-MZ-2, and changes in the expression levels of the genes involved in PTS were similar to the results obtained by Larsen et al. (2006). However, in the PTS, the carbohydrates are phosphorylated at the expense of PEP as the product that is transported across the cell membrane (Jojima et al., 2010). PEP can be rapidly used for the growth of the bacteria at the exponential growth phase. The limitation of phosphorylated substrates might lead to a significant decrease of the transcription levels of the genes involved in PTS at the mid-exponential growth phase of ST-MZ-2. Moreover, the rapid consumption of intracellular nutrients at the exponential growth phase led to the intake of the extracellular nutrients and induced the high expression levels of the genes at the end-exponential growth phase. LAB transport glucose using the mannose-PTS system, the glucose-PTS system, and the action of a permease (Rio et al., 2015). The present results showed that *manXY* and *crr* involved in these systems had high expression levels. The extracellular glucose was decreased significantly at the end-exponential growth phase compared with the mid-exponential growth phase. Lactose and galactose were transported by the LacS permease (Barrangou et al., 2006; Jojima et al., 2010). *LacS* had a similar trend of expression as the genes involved in PTS. Consequently, the change in the concentration of extracellular galactose was similar to that of the concentration of extracellular glucose from the lag phase to the end-exponential growth phase. Furthermore, *S*. *thermophilus* encounters many stress factors simultaneously at the end-exponential growth phase, such as low intracellular acidity and high extracellular osmotic pressure (Shan et al., 2016; Zhengwen et al., 2017). Stress factors inhibit the growth and metabolic activities of LAB by affecting the expression of the genes and the uptake of nutrients. Thus, these reasons led to the low expression levels of most genes during the stationary phase.

The *galKTEM* and *lacSZ* genes are involved in galactose metabolism in the Leloir pathway in *S. thermophilus* (Vaillancourt et al., 2008). Most *S*. *thermophilus* cannot utilize galactose because genes involved in galactose metabolism had lower expression levels (De Vin et al., 2005). The inability of strain *S. thermophilus* SMQ-301 to grow on galactose results from a lack of galactokinase (encoded by *galK*), which transforms α-D-galactose into α-D-galactose-1-phosphate (Vaillancourt et al., 2008). The transformant of *S. thermophilus* SMQ-301 expressing *Streptococcus salivarius galK* and *galM* was able to grow on galactose and expelled at least twofold less galactose into the medium during growth on lactose (Vaillancourt et al., 2008). However, in this study, the expression levels of *galKTM* and *lacZ* were high during the exponential growth phase, which indicated that galactose might be utilized during the growth of ST-MZ-2. In addition, the change in the concentration of extracellular galactose indicated that galactose was reabsorbed into cell from the mid-exponential growth phase to the end-exponential growth phase and intracellular glucose might not meet the requirements for cell proliferation during this period. And the total concentration of galactose including extracellular galactose and intracellular galactose was decreased. This phenomenon also proved this point on the other hand.

Some LAB have the ability to change homofermentation to mixed-acid metabolism when growth rates and glycolytic flux are low (Zaunmüller et al., 2006). Pyruvate metabolism is mediated by LDH and pyruvate formate lyase (PFL) under substrate abundant or limitation (Gänzle, 2015). Lactic acid is the primary product when the substrate is abundant. During the glycolytic pathway, the activities of LDH, PFL and pyruvate dehydrogenase (PDH) were influenced by the NADH/NAD^+^ ratio (Kowalczyk and Bardowski, 2007). For the high glycolytic carbon flux, a high NADH/NAD^+^ ratio activated the activity of LDH and inhibited the activity of GADPH. The intracellular NADH/NAD^+^ ratio was highest at the lag phase during the culture of ST-MZ-2 (Liu et al., 2018). The results showed that the concentration of lactic acid increased rapidly and the high expression levels of related genes were detected from the end-lag phase. Interestingly, acetic acid and ethanol were also increased detected at the same time, and their concentrations were increased during the culture of ST-MZ-2. Furthermore, the related genes were also detected. This indicated that homolactic fermentation and mixed-acid fermentation might occur simultaneously during the culture of ST-MZ-2. The high concentration of lactic acid indicated that homolactic fermentation was the primary reaction.

*Tetragenococcus halophilus* converts citric acid via PFL to acetic acid and ethanol, and numerous LAB have the ability to convert citric acid to lactate (Gänzle, 2015). In this study, the concentration of citric acid decreased quickly at the mid-exponential growth phase. And *pfl* had a high expression level during the lag phase. Thus, we deduced that citric acid might be metabolized and utilized by ST-MZ-2. Acetic acid interferes with bacterial energetics, inhibits the expression of the proteins and genes involved in the stress response and regulation processes, causes low pH, and inhibits bacterial growth (Papagianni, 2012). Pyruvate carboxylase could decrease acetic acid by 60% and increase protein yield by 68% in *Escherichia coli* (March et al., 2002). In this study, the concentration of intracellular acetic acid was the highest at the mid-exponential growth phase. The concentration of extracellular acetic acid was increasing during the culture and the concentration of total acetic acid was maintained at a stable level. Bacteria can deploy various mechanisms to combat environmental stresses (Van et al., 2017). ST-MZ-2 expelled acetic acid and degraded the intracellular acetic acid to response to high concentration of acetic acid. According to the high expression of *pyc*, we deduced that part of the extracellular acetic acid was neutralized by the added NaOH and part of the intracellular acetic acid might be degraded pyruvate carboxylase.

Proteins involved in the glycolytic pathway, such as PYK, GAPDH, and phosphoglycerate kinase (PGK), were highly induced during the lag phase (Larsen et al., 2006). The results showed that most of the genes involved in the carbohydrate metabolism had their highest expression levels at the lag phase. Genes, such as *ldh* and *pfl*, had high expression levels at the end-lag phase, while the metabolites increased significantly at the mid-exponential growth phase. The hysteresis of the transcriptional regulation of the genes might result in these results (Mayo et al., 2015). In addition, *pfk*, *gapA*, *ldh*, and *fbaA* were upregulated at the stationary phase, while the biomass did not increase, and the decrease in pH was also slow. The energy produced by carbohydrate metabolism might be primarily used to resist high osmotic pressure stress and intracellular acid stress.

## Conclusion

This study revealed changes in the expression level of the genes involved in carbohydrate transport and metabolism and changes in the concentration of carbohydrate metabolites in ST-MZ-2 during pH-controlled batch fermentations. Most genes involved in carbohydrate transport and carbohydrate metabolism had their highest expression levels at the end-lag phase. ST-MZ-2 had high expression levels of *LacZ* and *galKTM* and reabsorbed extracellular galactose into cell from the mid-exponential growth phase to the end-exponential growth phase showed that ST-MZ-2 metabolized galactose during the culture. In addition, we deduced that homolactic fermentation and mixed-acid fermentation might occur simultaneously during the culture of ST-MZ-2 and homolactic fermentation was the primary reaction. This will be our future research direction.

## Data Availability Statement

All datasets generated for this study are included in the article/Supplementary Material.

## Author Contributions

GL, AL, and ZF designed the study and the experiments. GL, YQ, JS, and YZ performed the experiments. GL, YQ, CL, and HC analyzed and interpreted the data. GL wrote the manuscript with support from ZF and XF. All authors reviewed the manuscript.

## Conflict of Interest

The authors declare that the research was conducted in the absence of any commercial or financial relationships that could be construed as a potential conflict of interest.

## Footnotes

1 http://www.genome.jp/kegg

2 http://www.ncbi.nlm.nih.gov/COG/
