# Supplementary material for: Metabolic Profiles of Carbohydrates in Streptococcus thermophilus During pH-Controlled Batch Fermentation
Source: Front Microbiol. 2020 May 29;11:1131. doi: 10.3389/fmicb.2020.01131 (PMC7272703; doi:10.3389/fmicb.2020.01131)
Supplement: Supplementary file 1 [file Table_1.DOCX]

**Supplementary data Table S1.** A list of primers used in this study

F, forward primer; R, reverse primer

| Gene | Gene description | Primer sequence (5’→3’) |
| --- | --- | --- |
| pgm | Phosphoglucomutase | F-TCCATCCTAGCCACCCGCAAG  R-GCCCTCAGCTTCCACCTCCTC |
| galT | UDP glucose – hexose – 1 – phosphate - uridine-based invertase | F-CGACAAGGGCGACGGCAAG  R-GCGGCGGAACGAGAAGAACTG |
| galK | Galactokinase | F-GCTCAAGGAGACGCTGGATGC  R-CGAACGCACGCACGAAGGAG |
| pgi | Glucose-6-phosphate isomerase | F-TGCCAACAAGGACCGCTTCAAC  R-GTCCACCAGCATCCGCATCAC |
| ldh | L-lactate dehydrogenase | F-TTGGGCAAAGGCTTGGCATCC  R-CTTCAGAGGGACGCCAGCAATG |
| lacZ | β-galactosidase | F-AAGCGGCCTGGACAAATGGAAG  R-TTTCGGAGCCACCAACTGCTTG |
| lacS | Lactose transporter | F-GCATCGTGGGCTTCGTGGTC  R-GGCGAGCGGCAGGTAGTAGG |
| pfk | Phosphofructokinase | F-GGCGACGCTCCTGGTATGAATG  R-TGATGCCGTATGCTTCCAACCC |
| glk | Glucokinase | F-GCTCTTGGCGAACGTTGGGTAG  R-CCAGCTCCGGCAACACCATG |
| pyc | Pyruvate carboxylase | F-TCTGCGAGGCGGCGATCTG  R-GTCCTTGACGGCGATGATGTGG |
| CcpA | Carbohydrate catabolic protein | F-GCGAAGCGGCGGTTTCAATG  R-AACCCTCGTGCAACTGCGTTAG |
| pyk | Pyruvate kinase | F-CGTTGCCGCTACCGCTTCTG  R-GCGTTCCGTGCGTTCATCAAAG |

Figure Legends

**Supplementary data Figure S1.** Trend graph of corresponding gene transcriptional expression by qRT-PCR analysis in ST-MZ-02 at T2, T3, and T4 compared with T1. The X axis displays the sampling time, and the Y axis displays the normalized fold expression of genes. The bars with different superscripts are significantly different (*P* < 0.05)

**Figure S1**
