# Supplementary material for: Metabolic Profiles of Carbohydrates in Streptococcus thermophilus During pH-Controlled Batch Fermentation
Source: Front Microbiol. 2020 May 29;11:1131. doi: 10.3389/fmicb.2020.01131 (PMC7272703; doi:10.3389/fmicb.2020.01131)
Supplement: Supplementary file 2 [file Table_2.DOCX]

**Supplementary data Table S2.** Composition of the complete chemically-defined medium

| Constituent | Concentration (g/L) | Constituent | Concentration (g/L) |
| --- | --- | --- | --- |
| Lactose | 10 | Adenine | 0.01 |
| Na-acetate | 1 | Guanine | 0.01 |
| NH_4_-citrate | 6 | Uracil | 0.01 |
| KH_2_PO_4_ | 3 | Xanthine | 0.01 |
| K_2_HPO_4_ | 2.5 |  |  |
|  |  | Aspartic acid | 0.46 |
| MgCl_2_.6H_2_O | 0.2 | Asparagine | 0.35 |
| CaCl_2_.2H_2_O | 0.05 | Glutamic acid | 0.40 |
| MnSO_4_.H_2_O | 0.028 | Glutamine | 0.39 |
| FeCl_2_.4H_2_O | 0.005 | Lysine | 0.44 |
| ZnSO_4_.7H_2_O | 0.005 | Arginine | 0.13 |
| CoCl_2_.6H_2_O | 0.0025 | Histidine | 0.15 |
| CuSO_4_.5H_2_O | 0.0001 | Proline | 0.68 |
|  |  | Phenylalanine | 0.28 |
| Ascorbic acid | 0.5 | Tryptophane | 0.05 |
| p-Amino benzoic acid | 0.01 | Methionine | 0.13 |
| Biotin | 0.01 | Alanine | 0.24 |
| 2-Deoxythymidine | 0.005 | Valine | 0.33 |
| Inosine | 0.005 | Leucine | 0.48 |
| Pyridoxamine-HCl | 0.005 | Isoleucine | 0.22 |
| DL-6,8-Thioctic acid | 0.0025 | Glycine | 0.18 |
| Pyridoxine-HCl | 0.002 | Serine | 0.34 |
| Nicotinic acid | 0.001 | Threonine | 0.23 |
| Riboflavin | 0.001 | Cysteine | 0.25 |
| Ca-pantothenate | 0.001 | Tyrosine | 0.29 |
| Thiamin-HCl | 0.001 |  |  |
| Folic acid | 0.001 |  |  |
| Cyanocobalamine | 0.001 |  |  |
